# Exploring cross-category relationships between symptoms in people with hypermobile EDS (hEDS) to identify disability patterns

**DOI:** 10.1371/journal.pone.0357565

**Published:** 2026-09-11

**Authors:** Jennifer Andrews, Zachery Harkey, Sarah Mathena, Maureen Galindo, Sarrah Hannon, Rohith Jesudas, Christina M. Laukaitis

**Affiliations:** 1 ARID Lab, Department of Pediatrics, University of Arizona, Tucson, Arizona, United States of America; 2 Stephens Family Clinical Research Institute, Carle Health, Urbana, Illinois, United States of America; 3 Banner University of Arizona Medical Center, Tucson, Arizona, United States of America; 4 Department of Hematology, St. Jude Research Hospital, Memphis, Tennessee, United States of America; 5 Carle Illinois College of Medicine, University of Illinois, Urbana, Illinois, United States of America; 6 Carl R. Woese Institute for Genomic Biology, University of Illinois, Urbana, Illinois, United States of America; Geisinger Health System, UNITED STATES OF AMERICA

## Abstract

**Background:**

Hypermobile Ehlers-Danlos Syndrome (hEDS) is a connective tissue disorder with variable symptom presentation across multiple organ systems and significant morbidity. Little is known about hEDS etiology and identifying patterns of symptom co-occurrence can reveal previously unidentified relationships between phenotypes and inform studies of underlying disease pathophysiology for symptoms that may share functional biological pathways. In this exploratory analysis, we specifically assessed the distribution of symptoms in case and controls to identify clusters of co-occurring symptoms.

**Methods:**

We have interrogated clinically relevant symptom areas in 47 females with hEDS, 36 age-matched female controls and 8 hypermobile patients without chronic pain. Studied symptoms include general health, mental health, body pain, vitality and energy, autonomic symptoms, bleeding, and gastrointestinal symptoms. We conducted hierarchal clustering on principle components (HCPC) to identify groups and compared the groups for the previously described symptoms. Radial plots were used to identify relationships between severe symptom categories.

**Results:**

Our analysis reveals statistically significantly more severe symptoms in all categories in people with hEDS compared with age- and sex-matched controls and asymptomatic hypermobile patients. HCPC identified clearly separated Low, Moderate, and High symptom groups within participants. The Low dysfunction groups include nearly all controls and hypermobile patients without chronic pain. The High dysfunction group includes ~60% of people with hEDS, while around 40% are in the Moderate dysfunction cluster. Cluster solutions for all participants were stable with moderate fit (silhouette 0.64; Jaccard boot mean 0.91). Group level radial plots showed high bleeding severity across all symptom clusters, while disproportional severity of general health, physical function, limitation of role due to physical symptoms, pain, and social functioning deficits differentiates the High from Moderate and Low Dysfunction clusters.

**Conclusion:**

Using this analysis at the group level has revealed patterns suggesting a progression of disease symptoms. People with hypermobility do not uniformly have severe symptoms but instead have some symptoms that differentiate from non-hypermobile individuals. While exploratory, using a radar multi-symptom analysis may be used to evaluate disproportionately severe symptoms contributing to the patterns of global symptom severity. These include pain but also ability to perform roles, suggesting strong utility of physical and occupational therapies to emphasize coping. This may also allow better targeting of etiological studies and may have additional utility at an individual level to develop symptom management strategies.

## Introduction

Ehlers-Danlos Syndromes (EDS) represent a group of 13 connective tissue diseases with overlapping signs and symptoms, 12 of which are associated with pathogenic variants in specific genes and with new gene associations ongoing [1,2]. The most common type is hypermobile EDS (hEDS), which has a wide spectrum of phenotypic presentations and the etiology remains poorly understood. There are currently no definitive biomarkers for hEDS and the diagnostic odyssey can take decades [3] but current prevalence estimates range from 1 in 500–1 in 5000 [4,5]. hEDS symptoms manifest as multisystemic dysfunction which causes significant morbidity in individuals.

While the relationship between hypermobility and pain symptoms has long been recognized [6], the variability of symptoms has complicated diagnosis, and this was compounded by lack of a diagnostic biomarker. For the purpose of recruiting a homogenous clinical cohort for gene-finding studies, an international consortium of clinicians developed the current diagnostic criteria [2]. The 2017 criteria for a diagnosis of hEDS include: 1) presence of generalized joint hypermobility measured using the 9-point Beighton scoring system (Criterion 1); 2) presence of 2 of 3 features (Criterion 2A – 2C); and 3) absence of evidence of a different EDS subtype, heritable or acquired connective tissue disorder or alternative diagnosis that explains the chronic pain or hypermobility (Criterion 3). Feature 2A is a list of 12 clinical signs in the skin, teeth, heart or visceral and pelvic organs, five of which are required to meet criteria. Feature 2B is a first degree relative who received an hEDS diagnosis using 2017 criteria and Feature 2C is a sequela of hypermobility occurring for more than 3 months: joint instability absent trauma; chronic pain in at least two limbs; or chronic widespread pain. Individuals who meet some but not all of Criteria 1 or 2 are considered to have a Hypermobility Spectrum Disorder (HSD) [7].

The 2017 Criteria sought to decrease reliance on acquired self-reported or observed symptoms, thus most of the dysfunctions experienced by patients are not included in the diagnostic criteria. A person with hEDS may experience symptoms of musculoskeletal pain, gastrointestinal pain and dysmotility, fatigue, headache, brain fog, dysautonomia (including postural orthostatic tachycardia syndrome), immune dysfunction implicating mast cells, interstitial cystitis, excessive bleeding and bruising, obstructive sleep apnea, pregnancy complications, and more [5].

HSD can be indistinguishable from hEDS in terms of an individual’s symptoms and reported functional impairments. Previous studies have asked whether specific symptoms might differentiate underlying differences between HSD or hEDS [8,9]. These studies found differences by age for skin-related objective signs and an overall increased severity of reported symptoms between clusters but the clusters did not clearly differentiate hEDS/HSD [8,9]. This contributes to the current hypothesis that hEDS/HSD and their associated morbidities are points across a severity spectrum, possibly representing earlier or later stages in disease progression rather than distinct diagnoses. We performed secondary analysis of data collected for a multiomics study that expands on the previous literature by comparing symptoms in affected individuals and their age- and gender-matched controls. Our aim is to describe the phenotypes of a diverse patient group to determine how symptoms and reported functional impairment could represent diagnostic or prognostic severity stages, building on the previous literature postulating an hEDS/HSD disease spectrum. Creating distinguishable clusters could provide better understanding for addressing the needs of those on hEDS/HSD spectrum based on the constellation and severity of symptoms they are experiencing rather than focusing on their diagnostic classification using the 2017 Criteria. These clusters can be used as a framework for evaluating multiomic data from patient samples and to individualize future treatment strategies.

## Materials and methods

### Participants

This study was performed under the supervision of the Carle Health Institutional Review Board (IRB; Protocol # 21CRU3373) and the University of Arizona IRB (Protocol # 1705446015). Individuals who were assigned female sex at birth aged 18−70 years were recruited for phenotypic and multiomic evaluation of hEDS in central Illinois and southern Arizona. Recruitment of participants in Illinois was performed through calls on social media through local patient support groups, fliers in Physical Therapy and Genetics clinics at Carle Health, emails sent to patients with clinical diagnoses made through Genetics at Carle Health, and the University of Illinois weekly campus informational electronic newsletter. Recruitment of affected participants in Arizona was done by contacting people previously taking part in the hEDS GENE study [9]. Recruitment of controls occurred at both locations using participant referral, social media and hospital study links. Controls were defined as individuals lacking chronic pain, generalized hypermobility and were age- (+/-3 years) and sex-matched to cases. All participants were evaluated by a clinician prior to enrollment using the hEDS 2017 Criteria performed specifically for the purpose of this study. We classified individuals who met full 2017 Criteria as hEDS Cases and individuals who met partial 2017 Criteria as “asymptomatic hypermobility” Cases. A trained research coordinator obtained written informed consent from each participant, with an independent witness. Recruitment occurred between 2/10/22 and 9/1/22.

### Questionnaires

Study participants completed 5 questionnaires (detailed below) describing current symptoms. Study data were collected and managed using the Research Electronic Data Capture (REDCap) hosted at the Carle Health and the University of Arizona administered via tablet at the intake visit [10]. Paper versions of the questionnaires were available to the participants upon request. The selected questionnaires investigate frequently reported complaints in hEDS/HSD and replicate questionnaires frequently used in hEDS/HSD research allowing for phenotype cross-compatibility in future meta-analyses.

#### Demographics.

A demographic questionnaire collected the participant’s age at enrollment and the 2017 Criteria from assessments performed by a trained clinician [2]. The 2017 Criteria diagnostic checklist yields a Beighton Score of generalized hypermobility (range: 0–9) and an eligibility cutoff of five for pubertal individuals 50 and younger and four for anyone over the age of 50 years. We collected presence of 12 objective signs and eligibility cutoff of five or more signs (Feature 2A); any first-degree family member diagnosed by the 2017 Criteria (Feature 2B); and chronic joint instability, widespread pain or pain in two or more limbs lasting three months or more (Feature 2C). See the 2017 Criteria diagnostic checklist on the Ehlers Danlos Society website (https://www.ehlers-danlos.com/wp-content/uploads/2017/05/hEDS-Dx-Criteria-checklist-1.pdf)

#### General Health.

The 36-Item Short Form Health Survey (SF-36) evaluates general health status [11]. It is composed of Likert scales varying in length from two to six response items. Higher scores indicate better health. It consists of the following subscales: physical functioning, role limitations due to physical health, role limitations due to emotional problems, energy/fatigue, emotional well-being, social functioning, pain, and general health. This has been used previously for exploration of symptoms in hypermobility [9,12–14].

#### Autonomic function.

We used the 31-question version of the Composite Autonomic Symptom Score (COMPASS31) [15]. The questionnaire yields six autonomic function subscales for orthostatic intolerance and vasomotor, secretomotor, gastrointestinal, bladder, pupillomotor, and an overall autonomic function score. The scale consists of 31 questions that are answered with Likert scales ranging from two to six items. Higher scores indicate increased severity, and the weighted subscales sum to the overall score of 100. This has been used in numerous studies of people with hypermobility [9,14,16].

#### Gastrointestinal Health.

The Birmingham Irritable Bowel Syndrome (IBS) scale has 11 questions reporting on frequency of symptoms on a scale of zero (none of the time) to five (all of the time) for items derived from the ROME II criteria [17]. Scores are summed to produce three subscales and a total score. The pain and constipation subscales consist of three questions each (range 0–15) and five questions for diarrhea (range 0–25) with a total score ranging from 0–55. This was performed as a pilot, so was completed only by participants recruited in Illinois. This has been used previously to evaluate symptoms in people with pelvic floor and functional gastrointestinal symptoms [18–20].

#### Bleeding Tendency.

Participants completed the self-administered version of the International Society for Thrombosis and Hemostasis Bleeding Assessment Tool (self-BAT) to document bleeding and bruising severity [21]. This consists of a screening questionnaire with follow-up queries. Any participant who indicated bleeding or bruising in one of 13 areas then provided additional details about the bleeding or bruising they have experienced. Scores range from 0–3 are summed to produce a single total score ranging from 0–39. Versions of this have been used to study people with hypermobility [9,22,23].

### Analysis

This exploratory analysis assessed the distribution of symptoms in case and controls using: 1) Hierarchical Clustering on Principal Components (HCPC); 2) Radar charts of subscale scores in the identified clusters; and 3) logistic regression comparing cases and controls. Statistical evaluations were performed using R version 4.3.2 [24] and the following additional packages cowplot version 1.1.3, gridExtra version 2.3, and factoextra version 1.0.7.

#### Hierarchical Clustering on Principal Components (HCPC).

We performed Hierarchical Clustering on Principal Components (HCPC) using FactoMineR version 2.11 [25]. HCPC combines the strengths of principal components analysis (PCA), hierarchical clustering and partition clustering methods into a single multivariate approach. Using HCPC is useful when there are numerous continuous variables with potential high correlation. The three methods are used in succession beginning with the dimension reducing PCA which removes noise from all variables and reduces the data into fewer dimensions to create more stable clusters [26]. We used the missMDA package in R to estimate the number of dimensions for PCA by cross validation. Symptom correlations are reported in S2 File and highly correlated variables are bound onto the same component using PCA, which is a mechanism of the method. Next, hierarchical clustering is performed using the Ward’s method. which is used when data are multidimensional such as in PCA. This determines the optimal number of clusters using a hierarchical tree which is visualized with a dendrogram and cluster mapping. Lastly a partition clustering is run using the optimal number of clusters identified in hierarchical clustering [27,28].

Given many of the scales have overlapping concepts (well-being, pain, gastrointestinal issues) and an extensive number of subscales representing the multidimensional aspect of symptom presentation in this population, HCPC is the most effective approach to generating a parsimonious approach to cluster identification in hEDS. Sufficient power in cluster outcomes is achieved by a large cluster separation (Δ = 4) using Euclidean distance calculations with as few as 20 individuals per cluster [29]. Missing scores were imputed for controls and cases separately using regularized multiple imputation using missMDA version 1.19 prior to conducting the principal components analysis [30].

We provide a comparison of age, diagnosis grouping and 2017 Criteria specifics for each cluster as well as the distribution of symptom scale scores that were used to generate the cluster solutions. Post-hoc descriptive comparisons of identified clusters were performed using chi-square for categorical variables and t-test or ANOVA for continuous variables compareGroups version 4.7.2 which automatically adjusts the p-values when doing post-hoc pair-wise comparisons across more than two groups [31]. Clusters were named based on the visual appearance of increasing severity in the subscale means across the groups.

#### Radar Charts of Symptom Scores.

We standardized each scale score to reference a range of 0–100 with lower scores representing dysfunction across all scales. The COMPASS31 and self-BAT were reverse scored then standardized, and the Birmingham IBS scale was standardized. The SF-36 is already in this format. We calculated a cluster global average which took the mean of all the scales by cluster. These global averages were used to assess the overall point difference between paired clusters (i.e., Low – Moderate and Moderate – High). We then calculated how each cluster scale score compared with the cluster global average. We marked any scale scores that differed from the cluster global average by 15 points or more. Scores that were higher than the cluster global average represented higher than expected dysfunction whereas scores that were lower than the cluster global average represented lower than expected dysfunction for the cluster.

#### Comparing cases and controls.

We are performing exploratory analysis on a dataset collected for a multiomics investigation. All cluster comparisons automatically include Bonferroni type correction as decribed by [32] which is embedded in the R compareGroups package.The data were not collected for the purposes of a phenotypic case-control study and are underpowered to perform traditional case-control analyses such as logistic regression. Therefore, we ran bivariate analysis to calculate odds ratios and provide confidence intervals for each SF-36 and COMPASS31 scale score using only the cases with age- and sex-matched controls.

## Results

**Fig 1:**
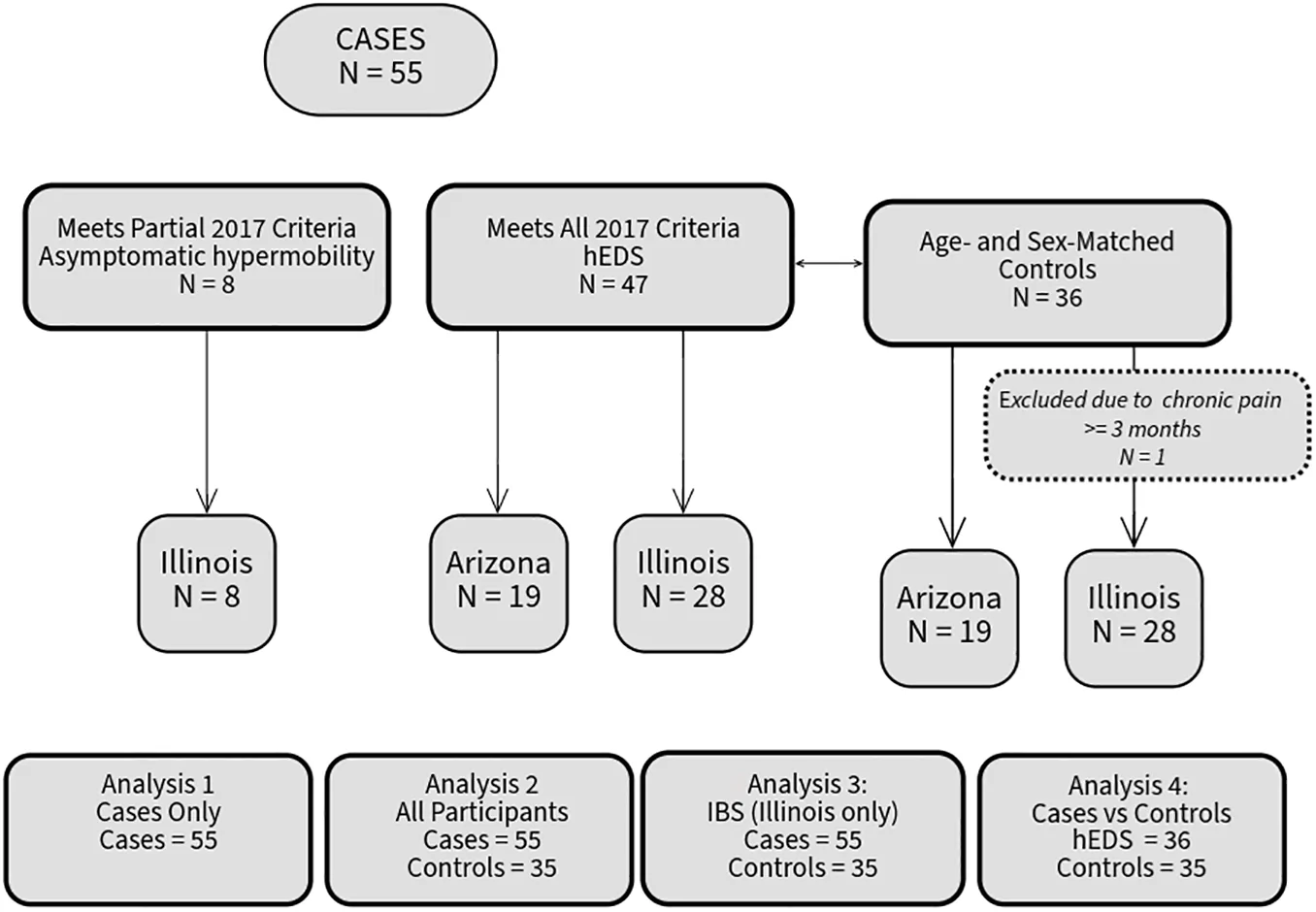
Enrollment flow chart. The count of cases and controls from Arizona and Illinois and the number of individuals included in each analysis are shown in a flow chart. Abbreviations: hEDS = hypermobile Ehlers Danlos Syndrome.

We enrolled 55 cases with a mean Beighton score of 7.2 and 36 age- and sex-matched controls without hypermobility (mean Beighton score 1.5). One control reported chronic pain lasting more than 3 months and was removed from the study (Fig 1). Of the 55 cases, 47 met full 2017 Criteria and are listed as hEDS and 8 met partial 2017 Criteria (no sequelae of hypermobility; less than 5/12 [2A] clinical signs) and are labeled ‘asymptomatic’ hypermobility (Table 1). We imputed missing data for the following variables: SF36 Average Change in Health for 3/55 cases, SF36 Average Change in Health for 5/35 controls, and SF36 Average Pain for 1 control. Three sets of HCPC analyses were performed, comparing symptoms across cases only, across cases and controls, and lastly the Birmingham IBS results in cases and controls for Illinois participants. We used the visual results of the radar charts to name the identified clusters.

**Table 1:** Demographic characteristics of cases and age-matched controls included in the study.

	Asymptomatic hypermobility (A)	Controls (C)	Hypermobile EDS (H)	p-value	A vs C	A vs H	C vs H
Total [N]	N = 8	N = 36	N = 47				
Demographics							
State [N, (%)]				0.07	0.13	0.12	0.67
Arizona	0 (0.00%)	12 (33.3%)	19 (40.4%)				
Illinois	8 (100%)	24 (66.7%)	28 (59.6%)				
Age at survey [M, (SD)]	28.9 (6.7)	44.7 (12.1)	42.1 (12.6)	0.01	0.00	0.01	0.61
2017 Diagnostic Criteria Checklist							
Criterion 1 – Generalized Joint Hypermobility [M, (SD)]	5.88 (1.46)	1.47 (1.46)	7.43 (1.35)	<0.001	<0.001	0.01	<0.001
Criterion 2 – Two or more Features Present [N, (%)]							
Feature A: At least 5 objective signs	1 (12.5%)	0 (0.00%)	44 (93.6%)	<0.001	0.18	<0.001	<0.001
Feature B: History of 1st^º^ relative by 2017 criteria	0 (0.00%)	0 (0.00%)	9 (19.1%)	0.01		0.33	0.01
Feature C: Pain or autraumatic instability	0 (0.00%)	1 (2.78%)	46 (97.9%)	<0.001	1.00	<0.001	<0.001
Objective signs [M, (SD)]	2.50 (2.00)	1.03 (0.97)	5.21 (0.75)	<0.001	0.00	<0.001	<0.001
Criterion 3 – Exclusion of skin fragility and alternate diagnoses	8 (100%)	36 (100%)	47 (100%)				
**Feature Specifics (A, B & C) [n, (%)]**							
Unusual soft skin	2 (25.0%)	6 (16.7%)	38 (80.9%)	<0.001	0.62	0.01	<0.001
Skin hyperextensibility	5 (62.5%)	11 (30.6%)	30 (63.8%)	0.01	0.18	1.00	0.02
Unexplained skin striae	1 (12.5%)	2 (5.56%)	29 (61.7%)	<0.001	0.46	0.03	0.001
Piezogenic papules	5 (62.5%)	12 (33.3%)	43 (91.5%)	<0.001	0.23	0.08	<0.001
Abdominal hernias	0 (0.00%)	0 (0.00%)	4 (8.51%)	0.20		1.00	0.26
Atrophic scars	1 (12.5%)	0 (0.00%)	28 (59.6%)	<0.001	0.18	0.03	<0.001
Organ prolapse	0 (0.00%)	0 (0.00%)	3 (6.38%)	0.44		1.00	0.51
Dental crowding/palate	2 (25.0%)	4 (11.1%)	31 (66.0%)	<0.001	0.30	0.07	<0.001
Arachnodactyly	3 (37.5%)	2 (5.56%)	26 (55.3%)	<0.001	0.05	0.46	<0.001
Arm span-to-height	1 (12.5%)	0 (0.00%)	4 (8.51%)	0.11	0.27	0.56	0.27
Mitral valve prolapse	0 (0.00%)	0 (0.00%)	6 (12.8%)	0.06		0.58	0.07
Aortic root dilation	0 (0.00%)	0 (0.00%)	3 (6.38%)	0.44		1.00	0.51
Family History	0 (0.00%)	0 (0.00%)	9 (19.1%)	0.01		0.33	0.01
2 + Limbs pain ≥ 3m	0 (0.00%)	1 (2.78%)	41 (87.2%)	<0.001	1.00	<0.001	<0.001
Widespread pain ≥ 3m	0 (0.00%)	1 (2.78%)	25 (53.2%)	<0.001	1.00	0.01	<0.001
Recurrent instability	0 (0.00%)	0 (0.00%)	14 (30.4%)	<0.001		0.10	0.00
Cluster Membership							
Cluster 1				<0.001		<0.001	
Low Dysfunction	8 (100%)		2 (4.26%)				
Moderate Dysfunction	0 (0.00%)		18 (38.3%)				
High Dysfunction	0 (0.00%)	.	27 (57.4%)				
Cluster 2				<0.001	0.084	<0.001	<0.001
Low Dysfunction	6 (75.0%)	34 (97.1%)	1 (2.13%)				
Moderate Dysfunction	2 (25.0%)	1 (2.86%)	18 (38.3%)				
High Dysfunction	0 (0.00%)	0 (0.00%)	28 (59.6%)				
Cluster 3				<0.001	0.006	0.040	<0.001
Low Dysfunction	1 (3.03%)	10 (43.5%)	0 (0.00%)				
Moderate Dysfunction	10 (62.5%)	13 (56.5%)	4 (16.0%)				
High Dysfunction – No Constipation	1 (12.5%)	0 (0.00%)	12 (48.0%)				
High Dysfunction – No Diarrhea	2 (25.0%)	0 (0.00%)	9 (36.0%)				

M: mean; SD: standard deviation.

### Comparison of cases only

**Fig 2:**
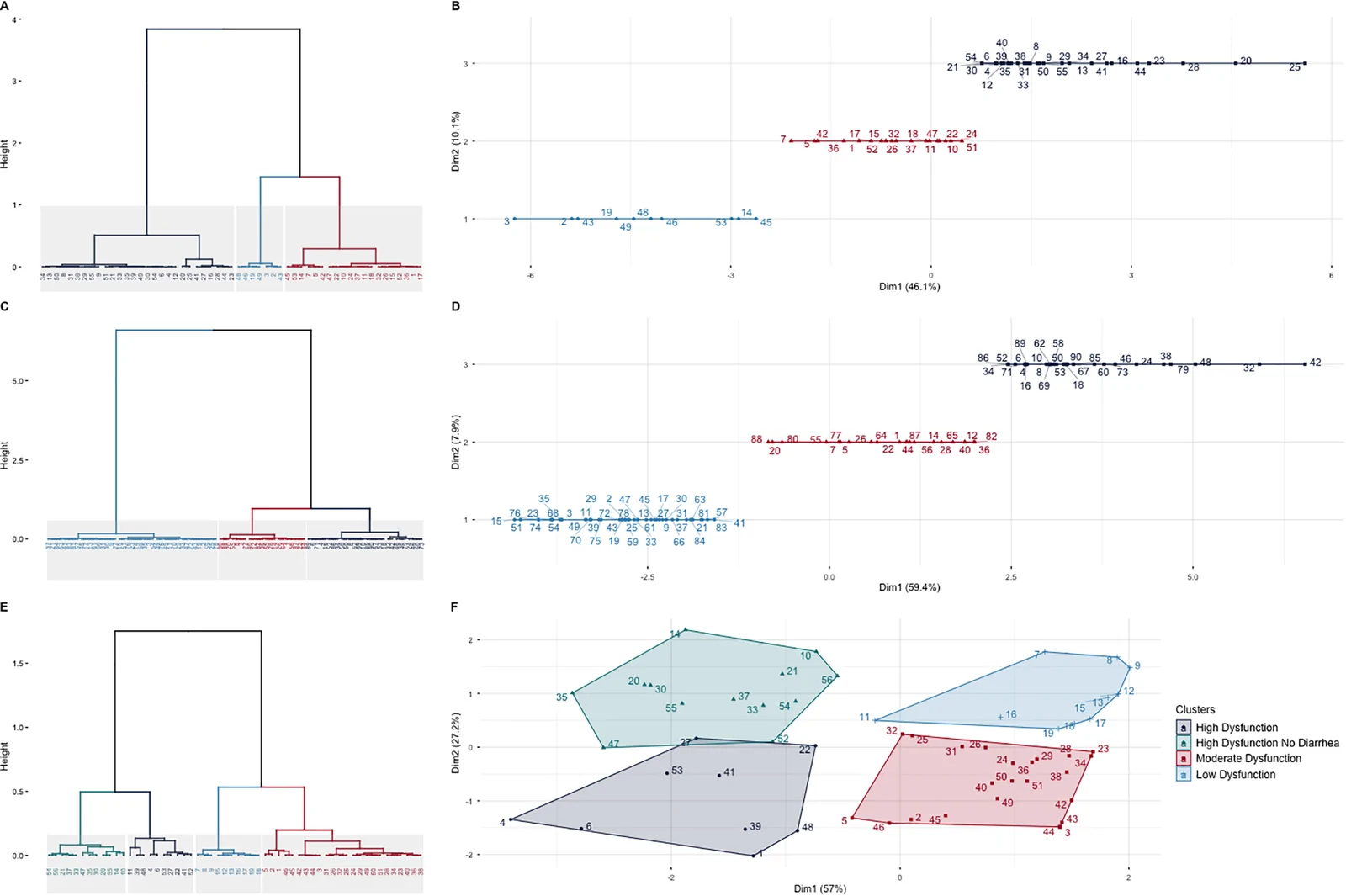
Dendogram (left) and clustering (right) resulting from the Cases Only (A, B), All Participants (C, D), and the IBS scale (E, F) analyses. Numbers represent the individual participants assigned to each cluster by type of participant (A = asymptomatic hypermobility, C = control, H = hEDS). Those in the Low dysfunction cluster (1) are shown in light blue. Those in the Moderate dysfunction cluster (2) are in red. Those in the High dysfunction cluster (3) are in dark blue. The cluster nomenclature and colors carry through the remaining figures and tables.

The Cases Only group included 55 cases and we clustered on the 17 scores included in the autonomic function (COMPASS31), bleeding (self-BAT) and general health (SF-36) scales. Diagnosis and Age at survey were include as supplemental qualitative and quantitative variables respectively in the PCA. The PCA reduced the scales to a single dimension (cumulative variance 46.1%) and HCPC cluster visualization demonstrates three distinct clusters (Fig 2A, B). Visual inspection of the dendrogram indicates the High Dysfunction group differentiates distinctly while the remaining individuals cluster into Low and Moderate Dysfunction groups. The cluster separation was intermediate (Δ = 3.4) but there are fewer than 20 participants in the Low Dysfunction group.

There were no significant differences between age of the groups, however the mean age does increase from Low to Moderate to High Dysfunction (Table 2). Low Dysfunction includes all the asymptomatic hypermobility cases (86%) and this correlates with their lower Beighton scores and numbers of objective signs. There were few differences in specific features other than a lower frequency of unusually soft skin and chronic pain in at least two limbs for more than three months in the Low Dysfunction cluster (Table 2).

**Table 2:** Cases only cluster comparisons of demographics, diagnostic criteria and self-reported symptom scales. Complete data are available in Supplemental Tables. Letters correspond to locations in the radar charts located in Fig 2 and -/ + indicates direction for low to high level of function for scale.

	Cluster by level of dysfunction						p-value	L vs M	L vs H	M vs H	Comp
	(L)ow		(M)oderate		(H)igh						
Total [N]	N=10		N=18		N=27						
Demographics											
State [N, (%)]							0.649				
Arizona	2	20%	7	39%	10	37%					
Illinois	8	80%	11	61%	17	63%					
Diagnosis [N, (%)]							<0.001	<0.001	<0.001	.	
Asymptomatic Hypermobility	8	80%	0	0%	0	0%					
Hypermobile Ehlers Danlos Syndrome	2	20%	18	100%	27	100%					
Age at survey [M, (SD)]	33.70	11.80	40.30	13.40	42.60	12.30	0.174				
2017 Diagnostic Criteria Checklist											
Criterion 1 – Generalized Joint Hypermobility											
Beighton Score [M, (SD)]	6.00	1.49	7.00	1.28	7.78	1.28	0.002	0.143	0.002	0.139	**L** to **H**
Criterion 2 – Two or more Features Present [N, (%)]											
Feature A: At least 5 objective signs	3	30%	17	94%	25	93%	<0.001	0.001	0.001	1.000	**L** to **MH**
Feature B: History of 1st^º^ relative by 2017 criteria	0	0%	3	17%	6	22%	0.315				
Feature C: Pain or atraumatic instability	2	20%	17	94%	27	100%	<0.001	<0.001	<0.001	0.400	**L** to **MH**
Objective signs [M, (SD)]	3.3	2.45	5.11	0.83	5.19	0.62	<0.001	0.001	<0.001	0.978	**L** to **MH**
Feature Specifics (A, B & C) [n, (%)]^1^											
Unusual soft skin	4	40%	13	72%	23	85%	0.029	0.187	0.035	0.449	**L** to **H**
Unexplained skin striae	2	20%	10	56%	18	67%	0.045	0.172	0.070	0.660	
Dental crowding/palate	3	30%	14	78%	16	59%	0.048	0.061	0.227	0.333	
2 + Limbs pain ≥ 3m	1	10%	16	89%	24	89%	<0.001	<0.001	<0.001	1.000	**L** to **MH**
Widespread pain ≥ 3m	1	10%	9	50%	15	56%	0.036	0.073	0.069	0.951	
Recurrent instability	1	10%	9	50%	4	15%	0.021	0.073	1.000	0.073	
Symptom Scales											
SF-36 [M, (SD)] (+)											
GH: General Health	72.00	18.00	33.60	17.40	19.30	12.80	<0.001	<0.001	<0.001	0.009	**ALL**
∆H: Change in Health	65.60	18.60	50.00	21.00	35.60	20.20	0.001	0.176	0.002	0.062	**L** to **H**
PF: Physical Functioning	92.50	10.10	57.80	17.20	40.60	19.10	<0.001	<0.001	<0.001	0.005	**ALL**
LP: Role Limit – Physical	85.00	31.60	22.20	22.50	6.48	11.20	<0.001	<0.001	0.000	0.033	**ALL**
LE: Role Limit – Emotional	60.00	34.40	57.40	42.50	43.20	39.00	0.366				
Pn: Pain	90.50	9.49	48.60	15.40	36.90	19.20	<0.001	<0.001	<0.001	0.062	**L** to **MH**
EF: Energy/Fatigue	53.00	20.20	31.90	16.20	13.70	14.40	<0.001	0.005	<0.001	0.001	**ALL**
WB: Well-being	71.20	17.40	68.20	15.80	54.10	24.10	0.029	0.929	0.073	0.071	
SF: Social Functioning	87.50	13.20	59.00	12.70	34.70	21.20	<0.001	<0.001	<0.001	<0.001	**ALL**
Self-BAT [M, (SD)] (+)											
BL: Total Bleeding Score	3.12	1.89	5.91	3.27	10.20	3.32	<0.001	0.138	<0.001	0.003	**LM** to **H**
COMPASS31 [M, (SD)] (-)											
AF: Autonomic Function	19.60	12.40	36.00	8.13	50.80	10.10	<0.001	<0.001	<0.001	<0.001	**ALL**
OI: Orthostatic Intolerance	10.40	9.47	16.90	6.52	21.80	7.95	0.001	0.098	0.001	0.109	**L** to **H**
VM: Vasomotor	0.50	1.05	1.16	1.34	2.50	1.06	<0.001	0.329	<0.001	0.001	**LM** to **H**
SM: Secretomotor	1.07	2.31	3.93	3.31	7.46	3.05	<0.001	0.052	<0.001	0.001	**LM** to **H**
BD: Bladder	0.44	0.57	1.73	1.22	2.67	1.67	<0.001	0.059	<0.001	0.074	**L** to **H**
PM: Pupillomotor	1.40	0.66	2.39	0.51	3.02	0.97	<0.001	0.007	<0.001	0.030	**ALL**
GI: Gastrointestinal	5.80	3.89	9.92	3.62	13.40	2.59	<0.001	0.006	<0.001	0.003	**ALL**

^1^Nonsignificant features removed: skin hyperextensibility; piezogenic papules; abdominal hernias; atrophic scars; organ prolapse; arachnodactyly; arm span-to-height ratio; mitral valve prolapse; aortic root dilation; family history.

M: mean; SD: standard deviation.

There were significant differences between the three clusters for scale scores used in clustering which increased in dysfunction from the Low to High clusters in 8 of 17 symptom subscales. Notably the SF-36 Pain subscale only differed in the Low cluster and there were no differences in Well-being and Role Limitations due to Emotional Health. Otherwise, the High Dysfunction group differentiated with more diminished capacity from either the Low or and Low and Moderate clusters (Table 2).

**Fig 3:**
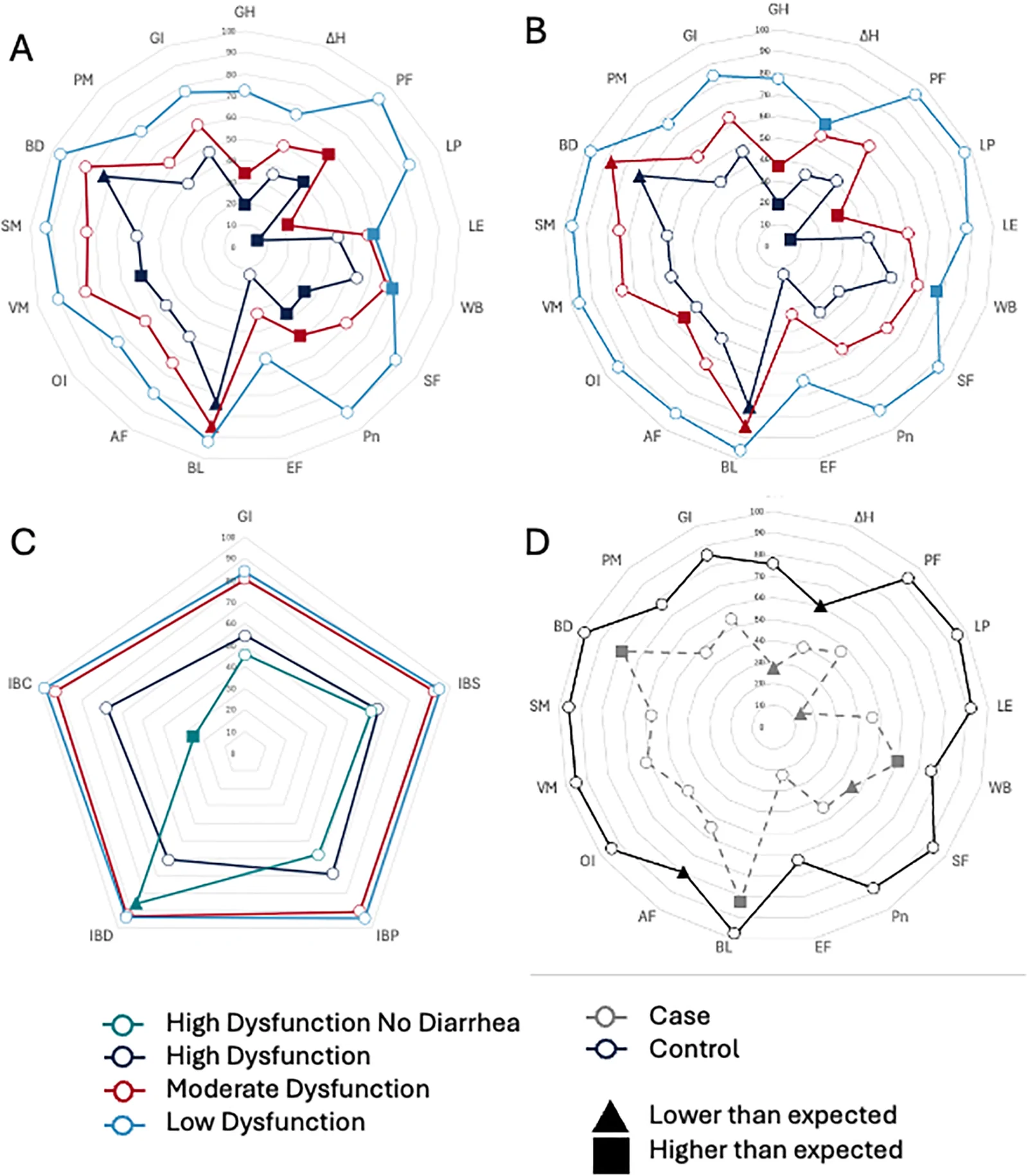
Radar charts with standardization for Cases Only (A), All Participants (B), IBS scale (C) and HEDS and Control (D) plotted separately. Essentially, severe symptoms plot closer to the center of the plot, while symptoms at the edges of the plot are consistent with improved functional status. In all three plots, the High Dysfunction cluster is on the inside in dark blue, with the lowest scores indicating the most dysfunction, while those on the outside are the Low Dysfunction cluster in light blue with the highest scores indicating the least dysfunction. Lower dysfunction than expected (p) reflects a scale score that 15 points or more above the cluster global average whereas Higher dysfunction than expected (¢) indicates a score that 15 points or more below the cluster global average. Abbreviations: GH – general health, ∆H – change in health, PF – physical functioning, LP – role limit – physical, LE – role limit – emotional, WB – well-being, SF – social functioning, Pn – pain, EF – energy/fatigue, BL – total bleeding score, AF – autonomic function, OI – orthostatic intolerance, VM – vasomotor, SM – secretomotor, BD – bladder, PM – pupillomotor, GI – gastrointestinal score, total, IBP – gastrointestinal pain, IBD – diarrhea, IBC – constipation, IBS – irritable bowel symptom score.

Visual inspection of the radar chart (Fig 3A) demonstrates a similar pattern of increasing dysfunction by cluster. The mean score difference between the Low and Moderate Dysfunction clusters was 21 points and 16 points between the Moderate and High Dysfunction. Unexpected differences in the Low Dysfunction cluster include reduced function in the SF-36 Role Limitations due to Emotional Health and Change in Health and Energy/Fatigue. Moderate and Severe groups both reported worse SF-36 General Health, Role Limitations due to Physical Health and Energy/Fatigue compared with cluster global average. Vasomotor and Secretomotor dysfunction is lower in the moderate group as compared with the cluster global average.

### Comparison of all participants

The PCA for All Participants was expanded from the initial analysis to include both cases and controls. The PCA reduced the scales to a single dimension (cumulative variance 59.4%) and three distinct clusters (Fig 2C, D). Visual inspection of the dendrogram indicates that the Low Dysfunction group differentiates distinctly, while remaining individuals cluster into high and Moderate Dysfunction groups. The cluster separation was lower (Δ = 3.2) but each cluster had at least 20 participants.

The Low Dysfunction group included only one hEDS case and had a lower Beighton score and objective signs from the Moderate and High groups, which did not differ. The majority of the specific features were also only different for the Low Dysfunction group (Table 3).

**Table 3:** All participant cluster comparisons of demographics, diagnostic criteria and self-reported symptom scales. Complete data are available in Supplemental Tables. Letters correspond to locations in the radar charts located in Fig 2 and -/ + indicates direction for low to high level of function for scale.

	Cluster by level of dysfunction						p-value	L vs M	L vs H	M vs H	Comp
	(L)ow		(M)oderate		(H)igh						
Total [n]	N=41		N=21		N=28						
Demographics											
State [N, (%)]							0.869				
Arizona	13	31.7%	8	38.1%	10	35.7%					
Illinois	28	68.3%	13	61.9%	18	64.3%					
Diagnosis [n, (%)]							<0.001	<0.001	<0.001	0.072	**L** to **MH**
Asymptomatic Hypermobility	6	14.6%	2	9.5%	0	0.0%					
Control	34	82.9%	1	4.8%	0	0.0%					
Hypermobile Ehlers Danlos Syndrome	1	2.4%	18	85.7%	28	100.0%					
Age at survey [M, (SD)]	42.6	13.1	40.7	13.0	42.2	12.1	0.858				
2017 Diagnostic Criteria Checklist											
Criterion 1 – Generalized Joint Hypermobility											
Beighton Score [M, (SD)]	2.1	2.1	6.8	1.5	7.8	1.3	<0.001	<0.001	<0.001	0.133	**L** to **MH**
Criterion 2 – Two or more Features Present [N, (%)]											
Feature A: At least 5 objective signs	1	2.44%	18	85.70%	26	92.90%	<0.001	0.001	0.001	1.000	**L** to **MH**
Feature B: History of 1st^º^ relative by 2017 criteria	0	0.0%	3	14.3%	6	21.4%	0.053				
Feature C: Pain or autraumatic instability	1	2.4%	17	81.0%	28	100.0%	<0.001	<0.001	<0.001	0.028	**ALL**
Objective signs [M, (SD)]	1.22	1.3	5	138.0%	5.18	0.61	<0.001	<0.001	<0.001	0.848	**L** to **MH**
Feature Specifics (A, B & C) [n, (%)]^1^											
Unusual soft skin	7	17.1%	15	71.4%	24	85.7%	<0.001	<0.001	<0.001	0.291	**L** to **MH**
Skin hyperextensibility	14	34.1%	14	66.7%	17	60.7%	0.021	0.080	0.080	0.898	**L** to **MH**
Unexplained skin striae	1	2.4%	11	52.4%	19	67.9%	<0.001	<0.001	<0.001	0.421	**L** to **MH**
Piezogenic papules	16	39.0%	17	81.0%	27	96.4%	<0.001	0.006	<0.001	0.150	**L** to **MH**
Atrophic scars	0	0.0%	11	52.4%	18	64.3%	<0.001	<0.001	<0.001	0.585	**L** to **MH**
Organ prolapse	0	0.0%	0	0.0%	3	10.7%	0.039		0.125	0.25	
Dental crowding/palate	6	14.6%	14	66.7%	17	60.7%	<0.001	<0.001	<0.001	0.898	**L** to **MH**
Arachnodactyly	4	9.8%	16	76.2%	11	39.3%	<0.001	<0.001	0.013	0.023	**ALL**
Arm span-to-height	0	0.0%	2	9.5%	3	10.7%	0.049	0.167	0.167	1.000	
Mitral valve prolapse	0	0.0%	2	9.5%	4	14.3%	0.023	0.167	0.071	0.688	
Family History	0	0.0%	3	14.3%	6	21.4%	0.003	0.053	0.009	0.714	**M** to **H**
2 + Limbs pain ≥ 3m	0	0.0%	16	76.2%	25	89.3%	<0.001	<0.001	<0.001	0.263	**L** to **MH**
Widespread pain ≥ 3m	0	0.0%	10	47.6%	15	53.6%	<0.001	<0.001	<0.001	0.902	**L** to **MH**
Recurrent instability	1	2.4%	8	38.1%	5	18.5%	<0.001	0.001	0.049	0.235	**L** to **MH**
Symptom Scales Used in Clustering											
SF-36 [M, (SD)] (+)											
GH: General Health	77.2	15.1	36.9	17.4	19.5	12.6	<0.001	<0.001	<0.001	<0.001	**ALL**
∆H: Change in Health	60.3	18.6	54.8	20.3	35.2	19.9	<0.001	0.565	<0.001	0.003	**LM** to **H**
PF: Physical Functioning	94.6	9.8	62.4	18.3	40.4	18.8	<0.001	<0.001	<0.001	<0.001	**ALL**
LP: Role Limit – Physical	96.3	9.0	31.0	30.5	6.3	11.0	<0.001	<0.001	<0.001	<0.001	**ALL**
LE: Role Limit – Emotional	87.8	24.4	60.3	38.9	41.7	39.2	<0.001	0.007	<0.001	0.131	**L** to **MH**
Pn: Pain	89.4	10.9	56.1	19.0	36.3	19.1	<0.001	<0.001	<0.001	<0.001	**ALL**
EF: Energy/Fatigue	63.8	14.7	32.6	15.2	13.8	14.1	<0.001	<0.001	<0.001	<0.001	**ALL**
WB: Well-being	76.4	13.9	67.2	16.1	54.4	23.8	<0.001	0.146	<0.001	0.041	**LM** to **H**
SF: Social Functioning	93.3	11.6	63.1	16.0	35.3	21.0	<0.001	<0.001	<0.001	<0.001	**ALL**
Self-BAT [M, (SD)] (+)											
BL: Total Bleeding Score	1.5	1.6	5.9	2.9	9.7	3.9	<0.001	<0.001	<0.001	0.001	**ALL**
COMPASS31 [M, (SD)] (-)											
AF: Autonomic Function	8.9	7.5	35.7	7.7	49.7	11.5	<0.001	<0.001	<0.001	<0.001	**ALL**
OI: Orthostatic Intolerance	2.7	5.4	17.9	5.0	21.0	8.8	<0.001	<0.001	<0.001	0.239	**L** to **MH**
VM: Vasomotor	0.2	0.7	1.2	1.3	2.4	1.1	<0.001	0.001	<0.001	<0.001	**ALL**
SM: Secretomotor	0.6	1.4	3.9	3.2	7.2	3.3	<0.001	<0.001	<0.001	<0.001	**ALL**
BD: Bladder	0.3	0.5	1.3	1.1	2.7	1.7	<0.001	0.002	<0.001	<0.001	**ALL**
PM: Pupillomotor	1.2	0.9	2.2	0.6	3.0	1.0	<0.001	<0.001	<0.001	0.004	**ALL**
GI: Gastrointestinal	3.9	2.6	9.1	4.2	13.3	2.6	<0.001	<0.001	<0.001	<0.001	**ALL**

^1^Nonsignificant features removed: abdominal hernias; aortic root dilation.

M: mean; SD: standard deviation.

There were significant differences between the three clusters for scale scores increasing in dysfunction from the Low to High clusters in 13 of 17 symptom subscales. The SF-36 General Health and Energy/Fatigue subscales only differed in the High Dysfunction group, the SF-36 Role Limitations due to Physical Health only differed between Low and High Dysfunction groups, and the COMPASS31 Orthostatic Intolerance only differed for the Low Dysfunction cluster (Table 3).

Visual inspection of the radar chart (Fig 3B) repeats the pattern of increasing dysfunction by cluster. The mean score difference between the Low and Moderate Dysfunction clusters was 26 points and 18 points between the Moderate and High Dysfunction. Unexpected differences in the Low Dysfunction cluster include reduced function in the SF-36 Change in Health scale and Energy/Fatigue subscales. Moderate and Severe groups both demonstrate reduced SF-36 General Health, Role Limitations due to Physical Health, and Energy/Fatigue scores as compared with their cluster global average. This is very similar to the Cases Only (Fig 3A) radial graph.

### Birmingham IBS scale

The Birmingham IBS assessment included the 36 cases and 23 controls from Illinois. We clustered on four scores: the COMPASS31 gastrointestinal subscale plus the total, pain, diarrhea and constipation subscales included in the Birmingham IBS. Diagnosis and Age at survey were included as supplemental qualitative and quantitative variables respectively in the PCA. The PCA recommended five components but only four were included indicating four clusters (Fig 2E, F). The cluster separation was low in this analysis (Δ = 2.6) and there are fewer than 20 participants in multiple groups. This cluster has the fewest variables and the smallest n, providing an unstable solution and data. These are presented here solely to provide guidance in future analyses. Complete data are available in the supplemental materials (S1 File). The Low Dysfunction group was older and no controls appeared in either High Dysfunction group, leading to higher Beighton and objective signs scores (Table 4). The High Dysfunction group was the youngest and the Low Dysfunction group had the oldest mean age. No controls were assigned to either High Dysfunction cluster. Scaled scores in the Low-Moderate and High-High no Diarrhea groups were similar.

**Table 4:** Cluster comparisons of diagnostic criteria and self-reported symptom scales for the Birmingham IBS and COMPASS31 Gastrointestinal Subscale. Complete data are available in Supplemental Tables. Letters correspond to locations in the radar charts located in Fig 2 and/ + indicates direction for low to high level of function for scale.

	Cluster by level of dysfunction								p-value
	(H)igh		High no (D)iarrhea		(M)oderate		(L)ow		
Total [N]	1 N = 9		2 N = 13		3 N = 23		4 N = 11		
Age at survey [M, (SD)]	29.4	(7.3)	40.0	(6.6)	31.2	(6.7)	51.1	(5.5	<0.001
Diagnosis [N, (%)]									<0.001
Asymptomatic Hypermobility	1	11.1%	1	(7.7%)	6	(26.1%)	0	0.0%	
Control	0	0.0%	0	0.0%	13	(56.5%)	10	(90.9%)	
Hypermobile Ehlers Danlos Syndrome	8	(88.9%)	12	(92.3%)	4	(17.4%)	1	(9.1%)	
2017 Diagnostic Criteria Checklist									
Criterion 1 – Beighton Score [M, (SD)]	7.8	(1.5)	7.3	(1.3)	4.1	(2.1)	1.8	(2.6)	<0.001
Criterion 2 – Two or more Features Present									
Feature A: ≥ 5 objective signs [N, (%)]	8	(88.9%)	12	(92.3%)	4	(17.4%)	1	(9.1%)	<0.001
Feature B: 1^st^ degree relative	2	(22.2%)	1	(7.7%)	0	0.0%	0	0.0%	0.05
Feature C: Pain or autraumatic instability	8	(88.9%)	12	(92.3%)	4	(17.4%)	1	(9.1%)	<0.001
Objective signs [M, (SD)]	5.1	(0.8)	5.1	(1.1)	2.5	(1.7)	1.5	(1.3)	<0.001
Feature Specifics [n, (%)]									
Unusual soft skin	7	(77.8%)	9	(69.2%)	8	(34.8%)	3	(27.3%)	0.03
Skin hyperextensibility	8	(88.9%)	11	(84.6%)	13	(56.5%)	4	(36.4%)	0.03
Unexplained skin striae	5	(55.6%)	7	(53.8%)	3	(13.0%)	1	(9.1%)	0.01
Piezogenic papules	9	(100%)	12	(92.3%)	17	(73.9%)	4	(36.4%)	0.00
Atrophic scars	6	(66.7%)	10	(76.9%)	3	(13.0%)	0	0.0%	<0.001
Dental crowding/palate	3	(33.3%)	10	(76.9%)	6	(26.1%)	3	(27.3%)	0.02
2 + Limbs pain ≥ 3m	7	(77.8%)	11	(84.6%)	3	(13.0%)	1	(9.09%)	<0.001
Symptom Scales Used in Clustering									
COMPASS31 [M, (SD)] (-)									
GI: Gastrointestinal	11.4	(4.57)	13.6	(2.03)	4.93	(3.37)	4.06	(2.47)	<0.001
Birmingham [M, (SD)] (+)									
IBP: GI Pain	10.3	(2.40)	8.69	(2.43)	13.6	(1.59)	14.2	(0.87)	<0.001
IBD: Diarrhea	15.2	(2.64)	21.5	(2.22)	23.3	(1.84)	23.5	(2.70)	<0.001
IBC: Constipation	10.1	(3.62)	3.77	(2.35)	13.9	(1.63)	14.6	(0.67)	<0.001
IBS: IBS Score	35.7	(4.56)	33.9	(4.41)	50.7	(3.96)	52.3	(3.29)	<0.001

^1^Nonsignificant features removed: abdominal hernias; organ prolapse; arachnodactyly; arm span-to-height ratio; mitral valve prolapse; aortic root dilation; family history; recurrent instability; Widespread pain ≥ 3m.

M: mean; SD: standard deviation; EDS: Ehlers Danlos syndrome.

The radar chart for the Birmingham IBS scale is shown in Fig 3C. Here, there is a mean reduction in scale scores of only 3 points from Low to Moderate Dysfunction, but an average of 30 points difference from Moderate to both High Dysfunction groups. Overall, both High Dysfunction groups were similar, but those experiencing significant constipation issues reported minimal diarrhea symptoms as compared with their cluster global average.

For each analysis, HCPC selected the number of clusters using the relative loss-of-inertia criterion [33], yielding k = 3 for the case-only and full-sample analyses, and k = 4 for the IBS subset. As an independent check, we computed average silhouette width across candidate k = 2–6 for each analysis. For the Case Only and All Participant analyses, silhouette values were reasonable (0.51–0.70) and the chosen k was at the local silhouette maximum for Cases Only and close to the peak for All Participants (the silhouette favored a 2-cluster split). For the IBS subset, silhouette values were weak across all k values and we note that the IBS-specific clustering solution is reported here for information only and is not stable.

Cluster stability was assessed via nonparametric bootstrap resampling (B = 100), using the Jaccard bootstrap mean criterion [34] (High >0.85, Moderate 0.6–0.85, Low < 0.6 stability). The All Participant analysis (largest n, hree clusters of 21–41 participants) showed the strongest stability (Jaccard 0.85–0.98, dissolution ≤10%). The case-only analysis (three clusters of 10–27 participants) showed consistently moderate stability (Jaccard 0.66–0.77), with stability increasing alongside cluster size. This pattern indicates that cluster stability in our data is primarily a function of per-cluster sample size rather than case/control imbalance. We report the IBS subset clusters with corresponding caution and recommend they be interpreted as exploratory pending replication in a larger sample, while the All Participant and Case Only cluster solutions are better supported.

### Case-control comparison

**Fig 4:**
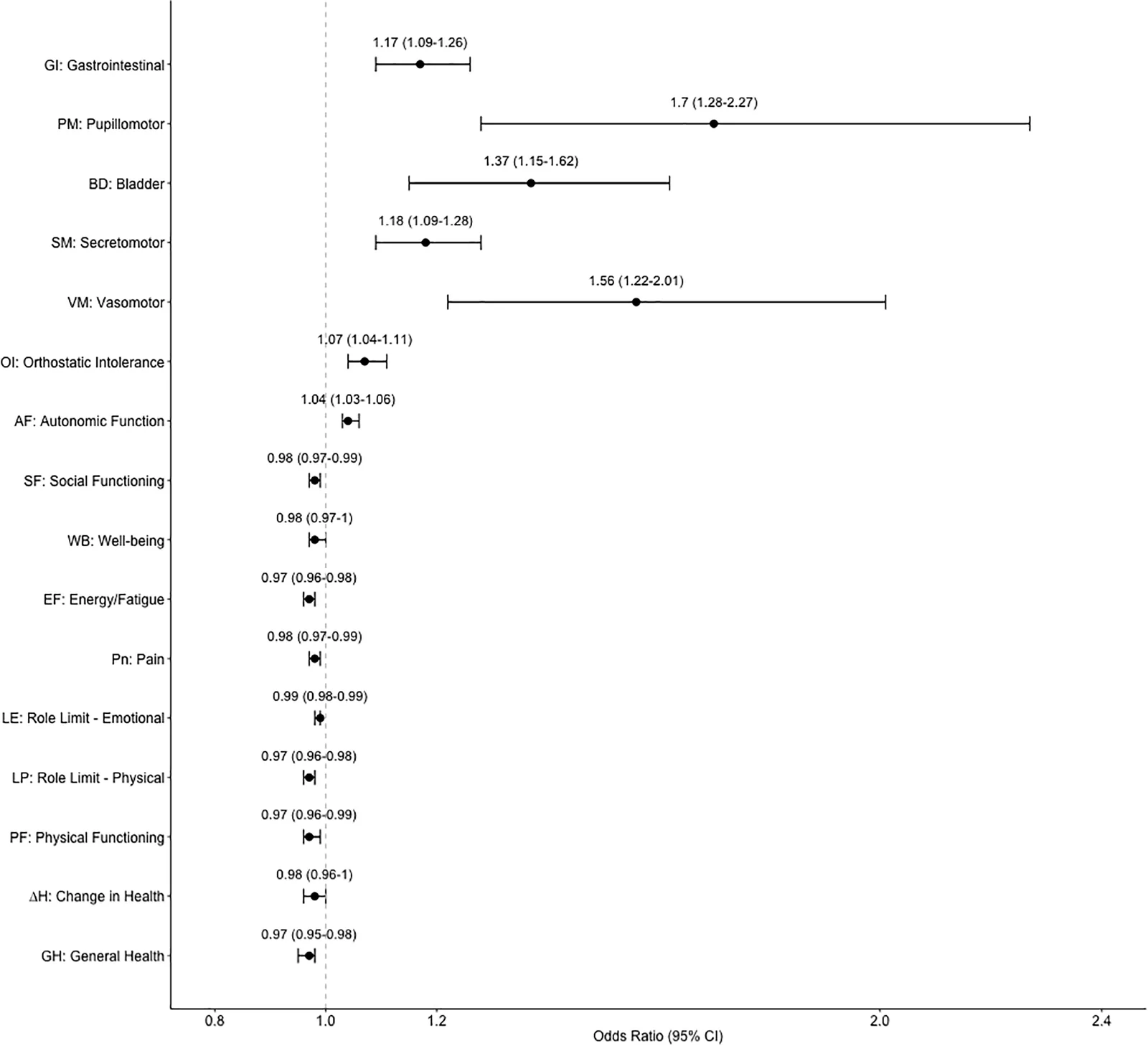
Bivariate odds ratios comparing cases and controls on the SF-36 and COMPASS-31 Subscales.

There were 36 cases and 35 age- and sex-matched controls. Bivariate odds ratios (Fig 4) demonstrate significant differences between cases and controls on all scales except the SF-36 Change in Health scale and Wellbeing subscales. The radar chart for is shown in Fig 3D. There is a mean reduction in scale scores of 38 points from control to case. As with the Cases Only and All Participants radar charts, the SF-36 General Health scale, Energy/Fatigue and Role Limitations due to Physical function subscales represent reduced function as compared with the case global average.

## Discussion

hEDS is part of a spectrum of symptomatic joint hypermobility conditions that represent the most common type of EDS and hypermobility-related disorders. There is a broad range of phenotypic variability present both within hEDS and across the HSD spectrum [35]. While new diagnostic criteria were created in 2017 [2], these were aimed at standardizing diagnosis and purposely avoided phenotypic inclusion. There have been cluster analyses performed previously [8,9,36,37]; however, our study is the first to compare phenotypic classification and clustering analyses on individuals with symptomatic and asymptomatic hypermobility compared with age- and sex-matched controls. Other reports have been case-only [8,36–38]. Some of these have studied patients evaluated prior to creation of the new diagnostic criteria [8,36] and one has relied solely on patient report of diagnosis and symptoms [37]. Our previous publication reported on non-hypermobile first-degree relatives of our affected patients [9], but did not have the increased range of including age- and sex-matched controls to allow for comparison to non-hypermobile individuals. Thus, past studies have been limited in both diagnostic uniformity and in the spectrum of participant severity.

Our data show similar trends to previous studies that reported a global increase in severity across multiple measures and body systems between clusters. Like Copetti et al (2019), we found a trend of global increase in severity of reported symptoms across our clusters and demonstrate a universal decline in health coupled with increased symptom severity reporting. We did not collect the Brief Pain Inventory (BPI), instead substituting the SF-36 Role Limitations due to Physical Function score standardized to a 10-point scale. This revealed an increase in severity score from 6.2 in the Low, 13 in the Moderate to 19 in the High Dysfunction groups [38]. The High Dysfunction group falls clearly in Copetti’s complex/severe cluster, while the Moderate Dysfunction group is on the border of their simple/milder cluster. The insightful Low Dysfunction cluster is unique to our study due to the inclusion of asymptomatic hypermobility and controls.

As with other clustering studies [8,9,36–38], participants were diagnosed based on their motivation to seek care and enrolled based on their ability to engage in research, which can be time consuming. Thus, enrollees have both enough disease burden to seek a diagnosis and enough time to participate in a study. It is well acknowledged that recruitment from clinics, support groups, and prior research cohorts likely enriches for more symptomatic individuals and creates the potential that cohorts bias toward severe symptomatology. While this study did not systematically address this potential source of bias, intentionally including people with fewer symptoms allows a more in-depth exploration of patterns of symptom co-occurrence, allowing us to develop hypotheses about symptom and progression. Ultimately, a longitudinal study of people ascertained solely on hypermobility may be useful to identify the triggers for symptomatic hypermobility.

Other authors have differentiated clusters based on disease burden using counts of morbidities [35,37]. De Wandele et al (2013) further demonstrated a positive correlation between increasing morbidity and pain and a negative correlation with function. In contrast, Schubart et al (2019) studied symptom clustering with a mixed sample of EDS subtypes and found a different composition of symptoms with a “Pain Dominant” subgroup who experienced high levels of pain but low levels of other included symptoms [36]. Their remaining two clusters follow a pattern of global increase in symptoms. We found similar trends in multisystem dysfunction correlating with pain and reduction in ability to function.

Overall, our exploratory data demonstrate trends and patterns similar to those previously identified in the literature, but the inclusion of controls and asymptomatic hypermobile individuals allows us to draw some additional conclusions regarding the composition of the clusters. First, overall assessments of General Health, Role Limitations due to Physical Dysfunction, Pain, and Social Functioning are impaired in all symptomatic individuals but disproportionately affect the High Dysfunction group. Second, there appears to be more severe vasomotor dysfunction reported in the High Dysfunction individuals. This is in keeping with our previous findings of increased autonomic dysfunction in hEDS versus HSD [9]. Lastly, both chronic diarrhea and constipation are more distressing in those severely affected which reflects the alternating bowel habits frequently cited in hEDS [39,40].

In contrast to the work of other groups [36,38], we identify a positive correlation between increasing Beighton score (Criterion 1 of the 2017 diagnostic criteria) and the number of objective signs (Criterion 2A of the 2017 diagnostic criteria) with clusters reporting increased symptom severity. At least in our cohort, symptom severity has a positive relationship to the severity of objective and observable patient features. We hypothesize that we are seeing a positive feedback loop where dysfunction in one system leads to disability globally.

Further study of hypermobile individuals with no chronic pain provides additional insights (Figure S1, Table S2 in S1 File). When compared with participants fully meeting hEDS criteria, individuals with hypermobility but no pain were much younger and had fewer objective signs; however, the only true post-hoc difference was in “pain in at least 2 limbs for more than 3 months.” The average difference in standardized scale scores between the two groups was 35 points and the mean scale scores for asymptomatic hypermobile individuals is 80. While the asymptomatic hypermobile group is relatively unaffected by physical pain or limitations, they show lower than expected scores in Change in Health, Emotional Well-being, Energy/Fatigue and Pupillomotor scales. We hypothesize that the pattern in this group is representative of the early stages of hEDS before the onset of chronic pain, limitations and additional objective signs. Alternatively, this group may provide clues as to resiliency, at least regarding pain. This group needs to be further investigated with a larger sample size.

Lastly, while frequent bruising and bleeding has long been recognized to be associated with symptomatic joint hypermobility diagnoses [9,41–44], the lack of evidence for clotting disorders or platelet dysfunction make it challenging to study bleeding in hEDS [41,42,45]. To our knowledge, this is the first study to include bleeding symptomatology in combination with other dysfunctions in phenotypic assessments of hEDS. Our data shows a significant increase in bleeding scores in cases, similar to the other symptoms studied. A self-BAT score ≥6 would trigger further clinical evaluation in a patient and both the Cases and High Dysfunction groups had average scores in that range (8.5 + /- 3.87; 10.2 + /-3.32, respectively). This supports previous reports that bleeding is an underrecognized issue in the hypermobile population [9,22,44]. The problem of bleeding and bruising is especially relevant in the setting of pregnancy and parity, frequent occurrences in female-predominant hEDS. Heavy menstrual [23,44] and peripartum [46,47] bleeding have both been reported at high frequency in people with hEDS and HSD. In addition, the hormonal and hemodynamic changes inherent to pregnancy can be triggers to symptom progression. This is clearly a question in need of further study.

Our study has several limitations, foremost being the low enrollment of controls to participants with hEDS and the exploratory use of an asymptomatic hypermobility arm. Most of our case participants cluster in the High dysfunction group. Rather than reflecting the typical frequency across the range of disease severity, this may reflect our recruitment strategy involving clinics and patient support groups, which are enriched for people with severe symptoms. Second, we have access to medical record data for many of these patients but a full abstraction for clinician-provided diagnoses has not yet been performed. Third, the scales used are standard scales for clinical research but were developed for more severely affected individuals and those with static, rather than fluctuating or functional disorders. For example, the autonomic function scale was developed for individuals with spinal cord injuries and the self-BAT is a screening tool for individuals with suspected inherited bleeding disorders. Scales with the ability to collect more nuanced differences between patients, and especially those with asymptomatic hypermobility, would yield significantly improved metrics to differentiate the individuals included in this study. While mitigated by the use of an age- and sex-matched control group, the use of self-reported symptom assessments can also be susceptible to biases such as recall bias, response bias, and social desirability bias. Fourth, while relevant to the disease state and improving power, including only individuals assigned female at birth limits generalizability. Fifth, the high bleeding scores, symptom heterogeneity, and questions of functional decline predominantly affecting females suggest a potential role of pregnancy in symptomatology [46,47]. This should be further explored. Sixth, information about psychosocial stressors, including environmental exposures, which can exacerbate underlying physiological dysfunction [48], was not systematically gathered. Finally, these findings are the result of exploratory analytic methods intended to guide future research and are not directly generalizable.

In short, individuals with symptomatic and asymptomatic hypermobility experience significant impairments in fulfilling their roles and their perceived overall health even when their symptoms are moderate. Moderately and severely affected individuals have an average impairment of 28% to 48% from the functional levels of their more typically functioning counterparts. Those differences are even more salient as they represent the average reduction in function across 17 morbidities and multiple body systems. These data will be coupled with ongoing multi-omics analysis to determine if cellular differences coincide with these phenotypic presentations.

## Supporting information

S1 File Data used for Figure 2; Expanded Tables 1–4 to include post-hoc comparison p-values; Complete data used for analyses; Data dictionary with variable definitions, types and missing counts. (XLSX)

S2 File Detailed methods for cluster solutions. (DOCX)
