# Supplementary material for: Exploring cross-category relationships between symptoms in people with hypermobile EDS (hEDS) to identify disability patterns
Source: PLoS One. 2026 Sep 11;21(9):e0357565. doi: 10.1371/journal.pone.0357565 (PMC13568515; doi:10.1371/journal.pone.0357565)
Supplement: Supplementary file 1 [file pone.0357565.s002.docx]

Supplemental Methods and results

1. Number of components estimated using estim_ncpPCA from the missMDA R package. No minimum or maximum values, default method (Regularized) and Kfold cross-validation specified. Number of comoponents proposed (ncp):
   a. Cluster 1 Cases = 1
   b. Cluster 2 All = 1
   c. Cluster 3 IBS = 5
2. Imputation performed using imputePCA from the missMDA R package. Ncp from step 1, default method (Regularized) and scale uniform weighting used.
3. Principal Component Analysis performed using PCA from the FactoMineR R package. Data with imputations from step 2 used imputer from step 1, default method (Regularized) and scale uniform weighting used. Diagnosis and Age at survey include as supplemental qualitative and quantitative variables respectively.

*Cluster 1: Cases Only*

eigenvalue	percentage of variance	cumulative percentage of variance

comp 1 6.45567414	46.1119582	46.11196

comp 2 1.41086418	10.0776013	56.18956

comp 3 1.09540271	7.8243051	64.01386

comp 4 1.03871540	7.4193957	71.43326

comp 5 0.87548540	6.2534671	77.68673

comp 6 0.67851781	4.8465558	82.53328

comp 7 0.62516066	4.4654333	86.99872

comp 8 0.53734075	3.8381482	90.83686

comp 9 0.37179600	2.6556857	93.49255

comp 10 0.32292746	2.3066247	95.79917

comp 11 0.21998439	1.5713171	97.37049

comp 12 0.15467359	1.1048114	98.47530

comp 13 0.13334128	0.9524377	99.42774

comp 14 0.08011624	0.5722588	100.00000

*Cluster 2: All (minus 1 control)*

eigenvalue	percentage of variance	cumulative percentage of variance

comp 1 8.31296977	59.3783555	59.37836

comp 2 1.10910780	7.9221986	67.30055

comp 3 0.86309705	6.1649790	73.46553

comp 4 0.81429360	5.8163828	79.28192

comp 5 0.67370766	4.8121976	84.09411

comp 6 0.55986653	3.9990467	88.09316

comp 7 0.44801760	3.2001257	91.29329

comp 8 0.37312097	2.6651498	93.95844

comp 9 0.24136727	1.7240519	95.68249

comp 10 0.21532841	1.5380601	97.22055

comp 11 0.12966231	0.9261594	98.14671

comp 12 0.10204751	0.7289108	98.87562

comp 13 0.09061656	0.6472612	99.52288

comp 14 0.06679694	0.4771210	100.00000

*Cluster 3: IBS scale participants*

eigenvalue	percentage of variance	cumulative percentage of variance

comp 1 2.28346086	57.0865214	57.08652

comp 2 1.07700273	26.9250682	84.01159

comp 3 0.61777451	15.4443626	99.45595

comp 4 0.02176191	0.5440478	100.00000

4. Symptom prevalence and centroid placement

*Cluster 1: Cases Only*

| Variable | Contribution | Correlation | Equal Share |
| --- | --- | --- | --- |
| completeObs.age | 1.9788378 | 0.3574176 | TRUE |
| completeObs.physical_functioning_avg | 10.1417409 | -0.8091463 | TRUE |
| completeObs.physical_role_limitations_avg | 9.8015283 | -0.7954588 | TRUE |
| completeObs.emotional_role_limitations_avg | 0.2359433 | -0.1234169 | TRUE |
| completeObs.pain_avg | 10.4815933 | -0.8225919 | TRUE |
| completeObs.energy_fatigue | 8.9831873 | -0.7615283 | TRUE |
| completeObs.emotional_wellbeing | 3.2683395 | -0.4593401 | TRUE |
| completeObs.social_functioning | 10.7435624 | -0.8328081 | FALSE |
| completeObs.vasomotor_weighted | 4.9030473 | 0.5626053 | TRUE |
| completeObs.secretomotor_weighted | 7.7140689 | 0.7056877 | TRUE |
| completeObs.gastrointestinal_weighted | 7.7512593 | 0.7073868 | TRUE |
| completeObs.bladder_weighted | 6.0449487 | 0.6246937 | TRUE |
| completeObs.pupillomotor_weighted | 6.9663086 | 0.6706133 | TRUE |
| completeObs.total_weighted | 10.9856346 | 0.8421382 | FALSE |

*Cluster 2: All (minus 1 control)*

| Variable | Contribution | Correlation | Equal Share |
| --- | --- | --- | --- |
| completeObs.age | 0.008159579 | 0.02604426 | TRUE |
| completeObs.physical_functioning_avg | 8.967832262 | -0.86341947 | TRUE |
| completeObs.physical_role_limitations_avg | 9.925907922 | -0.90837092 | TRUE |
| completeObs.emotional_role_limitations_avg | 3.257237042 | -0.52035866 | TRUE |
| completeObs.pain_avg | 9.578995416 | -0.89235587 | TRUE |
| completeObs.energy_fatigue | 9.078088002 | -0.86871095 | TRUE |
| completeObs.emotional_wellbeing | 3.881022800 | -0.56800374 | TRUE |
| completeObs.social_functioning | 9.729311860 | -0.89933017 | TRUE |
| completeObs.vasomotor_weighted | 5.269551462 | 0.66185816 | TRUE |
| completeObs.secretomotor_weighted | 7.324536524 | 0.78031180 | TRUE |
| completeObs.gastrointestinal_weighted | 8.767939013 | 0.85374242 | TRUE |
| completeObs.bladder_weighted | 6.689707009 | 0.74573006 | TRUE |
| completeObs.pupillomotor_weighted | 7.261765404 | 0.77696098 | TRUE |
| completeObs.total_weighted | 10.259945705 | 0.92352920 | TRUE |

*Cluster 3: IBS scale participants*

|  | Dim.1 | Dim.2 | Dim.3 | Dim.4 |
| --- | --- | --- | --- | --- |
| age | 3.013577 | 70.878040 | 26.05780606 | 0.05057687 |
| diarrhea_pt_sum | 24.469685 | 10.833892 | 52.75106499 | 11.94535807 |
| constipation_pt_sum | 30.017269 | 16.542425 | 21.10658722 | 32.33371823 |
| ibs_total_sum | 42.499469 | 1.745643 | 0.08454173 | 55.67034684 |

5. Symptom Correlations

*Cluster 1: Cases Only	Cluster 2: All (minus 1 control)*

6. Hierarchal Clustering on Principal Components performed using HCPC from the FactoMineR R package. Data from step 3 used with no pre-specified number of clusters suggested and automatic tree cut at suggested level.

HCPC Silhouette Comparison

| k | sil_case | sil_red | sil_ibs |
| --- | --- | --- | --- |
| 2 | 0.5049123 | **0.6918269** | 0.4123686 |
| 3 | **0.5464087** | **0.6441369** | 0.3446492 |
| 4 | 0.5363177 | 0.6167640 | 0.3490770 |
| 5 | 0.5799839 | 0.5084376 | 0.3425496 |
| 6 | **0.5862520** | 0.5483715 | 0.3417559 |

7. Cluster Stability

| Analysis | cluster | n | jaccard_bootmean | stability_rating | dissolved_pct |
| --- | --- | --- | --- | --- | --- |
| Case-only | 1 | 10 | 0.6624267 | Moderate | 39 |
| Case-only | 2 | 18 | 0.7005967 | Moderate | 36 |
| Case-only | 3 | 27 | 0.7706047 | Moderate | 28 |
| All participants | 1 | 41 | 0.8487776 | Moderate | 10 |
| All participants | 2 | 21 | 0.9793988 | Stable | 0 |
| All participants | 3 | 28 | 0.9123534 | Stable | 6 |
| IBS | 1 | 9 | 0.4827487 | Unstable | 64 |
| IBS | 2 | 13 | 0.7947625 | Moderate | 3 |
| IBS | 3 | 23 | 0.5622880 | Unstable | 50 |
| IBS | 4 | 11 | 0.5394168 | Unstable | 47 |

8. Univariate comparison of clusters performed using compareGroups from the compareGroups R package. Comparison statistics reported in manuscript Table 1 [10.18637/jss.v057.i12](https://doi.org/10.18637/jss.v057.i12) and reported briefly below:

|  | Table 1 | Tables 2 - 3 | |
| --- | --- | --- | --- |
|  | Overall P | Overall P | Pairwise P |
| Continuous normal | t.test | anova | TukeyHSD |
| Categorical | fisher.test | chisq.test | p.adjust using Benjamini & Hochberg (1995) |
